# Digital maturity and its determinants in General Practice: A cross-sectional study in 20 countries

**DOI:** 10.3389/fpubh.2022.962924

**Published:** 2023-01-13

**Authors:** Fábia Teixeira, Edmond Li, Liliana Laranjo, Claire Collins, Greg Irving, Maria Jose Fernandez, Josip Car, Mehmet Ungan, Davorina Petek, Robert Hoffman, Azeem Majeed, Katarzyna Nessler, Heidrun Lingner, Geronimo Jimenez, Ara Darzi, Cristina Jácome, Ana Luísa Neves

**Affiliations:** ^1^Faculty of Medicine of the University of Porto, Porto, Portugal; ^2^Institute of Global Health Innovation, Department of Surgery and Cancer, Faculty of Medicine, Imperial College London, London, United Kingdom; ^3^Westmead Applied Research Centre, Faculty of Medicine and Health, University of Sydney, Sydney, NSW, Australia; ^4^Australian Institute of Health Innovation, Macquarie University, Sydney, NSW, Australia; ^5^Irish College of General Practitioners, Dublin, Ireland; ^6^Health Research Institute, Edge Hill University, Ormskirk, United Kingdom; ^7^Galicia South Health Research Institute, Vigo, Spain; ^8^Leiro Health Center, Leiro, Spain; ^9^Center for Population Health Sciences, Lee Kong Chian School of Medicine, Nanyang Technological University, Singapore, Singapore; ^10^Department of Primary Care and Public Health, School of Public Health, Imperial College London, London, United Kingdom; ^11^Department of Family Medicine, Ankara University School of Medicine, Ankara, Türkiye; ^12^Department of Family Medicine, Faculty of Medicine, University of Ljubljana, Ljubljana, Slovenia; ^13^Department of Family Medicine, Sackler Faculty of Medicine, Tel Aviv University, Tel Aviv, Israel; ^14^Department of Family Medicine, Jagiellonian University Medical College, Kraków, Poland; ^15^Center for Public Health and Healthcare, German Center for Lung Research (DZL), Giessen, Germany; ^16^BREATH Hannover, Hannover Medical School, Hanover, Germany; ^17^Department of Public Health and Primary Care, Leiden University Medical Center, Leiden, Netherlands; ^18^CINTESIS@RISE, MEDCIDS, Faculty of Medicine of the University of Porto, Porto, Portugal

**Keywords:** primary care, quality of care, digital technology, digital maturity, electronic health records, health information interoperability

## Abstract

**Background:**

The extent to which digital technologies are employed to promote the delivery of high-quality healthcare is known as Digital Maturity. Individual and systemic digital maturity are both necessary to ensure a successful, scalable and sustainable digital transformation in healthcare. However, digital maturity in primary care has been scarcely evaluated.

**Objectives:**

This study assessed the digital maturity in General Practice (GP) globally and evaluated its association with participants' demographic characteristics, practice characteristics and features of Electronic Health Records (EHRs) use.

**Methods:**

GPs across 20 countries completed an online questionnaire between June and September 2020. Demographic data, practice characteristics, and features of EHRs use were collected. Digital maturity was evaluated through a framework based on usage, resources and abilities (divided in this study in its collective and individual components), interoperability, general evaluation methods and impact of digital technologies. Each dimension was rated as 1 or 0. The digital maturity score was calculated as the sum of the six dimensions and ranged between 0 to 6 (maximum digital maturity). Multivariable linear regression was used to model the total score, while multivariable logistic regression was used to model the probability of meeting each dimension of the score.

**Results:**

One thousand six hundred GPs (61% female, 68% Europeans) participated. GPs had a median digital maturity of 4 (P25–P75: 3–5). Positive associations with digital maturity were found with: male gender [*B* = 0.18 (95% CI 0.01; 0.36)], use of EHRs for longer periods [*B* = 0.45 (95% CI 0.35; 0.54)] and higher frequencies of access to EHRs [*B* = 0.33 (95% CI 0.17; 0.48)]. Practicing in a rural setting was negatively associated with digital maturity [*B* = −0.25 (95%CI −0.43; −0.08)]. Usage (90%) was the most acknowledged dimension while interoperability (47%) and use of best practice general evaluation methods (28%) were the least. Shorter durations of EHRs use were negatively associated with all digital maturity dimensions (aOR from 0.09 to 0.77).

**Conclusion:**

Our study demonstrated notable factors that impact digital maturity and exposed discrepancies in digital transformation across healthcare settings. It provides guidance for policymakers to develop more efficacious interventions to hasten the digital transformation of General Practice.

## Introduction

Digital technologies have revolutionized many aspects of modern society — health care is no exception (1). Around the world, the onset of the digital transformation has radically changed the primary care landscape (2, 3) through the widespread computerization and the digitalization of personal health information into Electronic Health Records (EHRs). Simultaneously, the dissemination of electronic medical devices (4), as well as adoption of systems enabling digital drug prescriptions, referrals, billing, scheduling tests and appointments are also major contributors to this change (5). Advances in digital technologies can also be seen from the proliferation of implantable devices which offer real time monitoring of physiological parameters (6, 7), to telemedicine (1) and mobile health—the use of mobile devices to improve health outcomes (8–11). This already ongoing digital transition has been further accelerated as a result of the COVID-19 pandemic (1, 12).

From facilitating communication between providers to improving prevention, achieving early diagnosis and providing timely treatments, digital technologies have demonstrated tremendous potential to improve health care delivery (13, 14). However, they are yet to play a major role among efforts to improve primary health care delivery (15). Nonetheless, the relevance of digital technologies keeps growing in primary care as governments' approaches to this sector continue to move toward the use of more collaborative systems (3). The extent to which digital technologies are employed to promote the delivery of high quality healthcare is known as digital maturity and it is an emerging concept across developed health care systems (16). The digital maturity of health professionals and systems is necessary to ensure a successful, scalable and sustainable digital transformation (17–19).

More than the modernization of medical resources, digital transformation is a complex multidimensional process (8) and therefore digital maturity, as any care intervention, needs to be rigorously evaluated and monitored to ensure successful implementation (16). While there have been studies focused on the assessment of digital maturity in secondary care (20, 21), similar efforts are likewise needed in primary care (3). The importance of exploring digital maturity shortcomings in primary care increases as some of its components such as EHRs have been cited as a contributor to physicians' burnout, particularly GPs' (22). To our best knowledge, digital maturity in primary care has not been previously evaluated.

This study assesses digital maturity — per individual dimensions (i.e., usage, resources and ability, interoperability, general evaluation methodology, and impact) and overall score (sum of all dimensions) — in General Practice across 20 countries. It also evaluates if the characteristics of participants or clinical practices, as well as features of EHR adoption, are associated with digital maturity. Our hypothesis is that the characteristics described above can affect digital maturity. The identification of such factors may contribute to developing more efficacious digital transformation implementation strategies worldwide.

## Methods

### Study design and setting

This is a cross-sectional study, utilizing an online questionnaire completed by GPs. Ethical approval was granted from the Imperial College Research Ethics Committee (Reference 20IC5956), which oversees health-related research with human participants. The study adheres to the STrengthening the Reporting of OBservational studies in Epidemiology (STROBE) guideline for cross-sectional studies. The research was conducted by a primary care consortium (inSIGHT Research Group) which gathers health professionals from 20 countries (Australia, Brazil, Canada, Chile, Colombia, Croatia, Finland, France, Germany, Ireland, Israel, Italy, Poland, Portugal, Slovenia, Spain, Sweden, Turkey, the United Kingdom, and the United States).

### Study population

Participants were eligible if they were GPs working in the countries above between March and September 2020.

### Sample size and recruitment

The sample size is superior to the total number of responses needed to provide a confidence level of 95% and a margin of error of 5% (901), according to the published protocol (23). Recruitment of participants was conducted by national leads who invited GPs working in their country to take part in the questionnaire *via* email and through social media channels, such as Facebook and LinkedIn. Participants were recruited between June and September 2020.

### Description of questionnaire

Investigators at the Patient Safety Translational Research Center and Department of Primary Care and Public Health at Imperial College London constructed the questionnaire. It was piloted by the national leads of the 20 inSIGHT Research Group associate countries in May 2020 and edited for national, cultural or organizational adaptations. The questionnaire was originally developed in English, and was translated to French, German, Italian, Portuguese, and Spanish by national leads to stimulate higher participation. The questionnaire was provided to participants through Qualtrics. The research protocol (including the full questionnaire) is available as a published paper in JMIR Research Protocols (23).

Demographic data (gender, age, and country), practice features (setting, number of hours of clinical work per week, number of years of experience as GP and involvement in teaching activities) and characteristics of access to EHRs (availability of EHRs, duration, and frequency of use) were collected. Digital maturity was assessed using the digital maturity framework developed by Flott et al. (Supplementary material), which considers the dimensions usage, resources and abilities (organizational and individual), interoperability, general evaluation methodology, and impact (21). These dimensions were assessed, respectively, by measuring agreement with the statements below.

- Usage: “*Most healthcare providers in our practice use the digital system*.”
- Resources and ability (organizational): “*Our organization is ready to use the digital system correctly*.”
- Resources and ability (individual): “*We have the individual abilities needed to use the digital system correctly*.”
- Interoperability: “*Our digital system has the capability to communicate across services or with other systems*.”
- General evaluation methodology: “*We have best practice digital maturity evaluation methods in place*.”
- Impact: “Our system has a positive impact in terms of outcomes for patients, structure, process or finance.”

All dimensions were evaluated by the participant as one of the following options: agree, neutral or disagree. The overall digital maturity score was calculated as the sum of the scores for the six dimensions. Whenever the participant expressed agreement with one dimension, one point was granted, and therefore overall digital maturity scores ranged between 0 to 6.

A full list of the questions included in this work is provided as Supplementary Table S1.

### Data analysis

All participants, even those in which some parameters were missing, were used in the analysis. Countries were categorized as European (Finland, France, Germany, Ireland, Italy, Poland, Portugal, Slovenia, Spain, Sweden, and the United Kingdom) and Non-European (remaining). The variable “Setting of practice” was split into “Rural” and “Urban.” The option “Prefer not to answer” in the questions regarding age, gender and involvement in teaching activities were treated as missing information.

**Figure 1:**
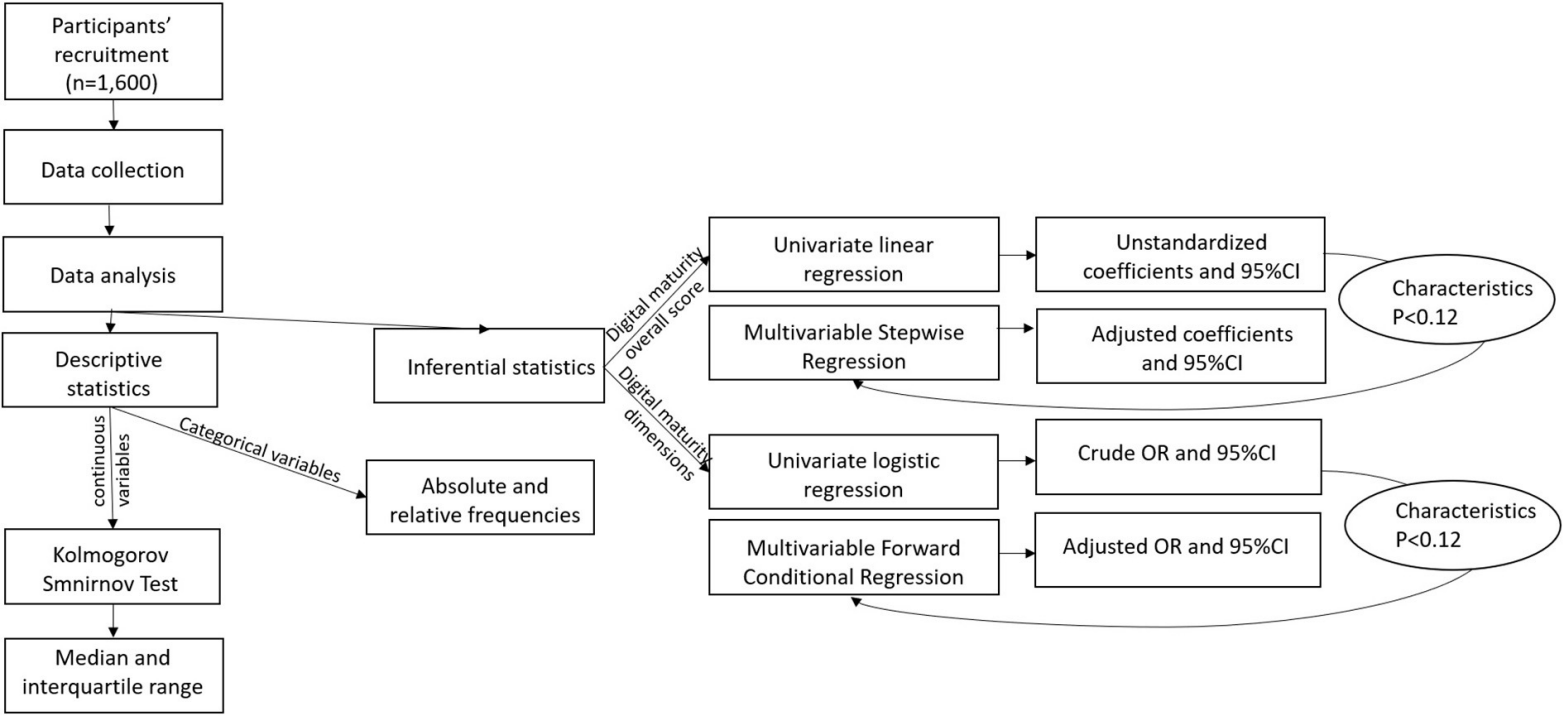
Visual representation of methodology. OR, odds ratio; CI, confidence interval; characteristics *P* < 0.12, independent variables whose association with the dependent variable had a *P*-value < 0.12.

The normality of distribution of each continuous variable was assessed using the Kolmogorov-Smirnov test (24). This test is one of the most general non-parametric methods for comparing two samples, as it is sensitive to differences in both location and shape of the empirical cumulative distribution functions of the two samples, therefore was chosen to assess normality in this case. Descriptive statistics were performed using absolute and relative frequencies for categorical variables, and median and interquartile range are presented for continuous variables with skewed distribution. Univariate linear regression was performed to determine the characteristics (i.e., gender, age, country, years of experience as GP, hours of clinical work per week, involvement in teaching activities, rural setting of practice, urban setting of practice, access to EHRs, duration and frequency of use of EHRs) associated with the digital maturity score (25) (continuous variable). Unstandardized coefficients (*B*) and 95% confidence intervals were calculated. All independent variables associated with digital maturity score with a *P*-value < 0.12 were included in the first multivariable model iteration. *P*-value represents the probability of obtaining the observed results, assuming that these characteristics were unrelated to the digital maturity score. The variables for multivariable analysis were chosen through the stepwise method. The models were evaluated using *P*-values, coefficients of determination (*R*^2^). Similarly, univariate binomial logistic regressions were used to identify characteristics possibly predicting the binomial outcome (0 = neutral/disagree, 1 = agree) of each of the six components of the digital maturity score usage, collective resources and ability, individual resources and ability, interoperability, general evaluation methods and impact (26, 27). Characteristics with *P*-value < 0.12 at univariate analysis were used in a multivariable logistic regression. The final model was obtained using a forward conditional regression. Adjusted odds ratio and 95% confidence intervals [aOR (95% CI)] were calculated (Figure 1). The models were evaluated using Hosmer Lemeshow tests and Nagelkerke's *R*-square (26, 27). Data were analyzed using IBM SPSS Statistics 26.0 (IBM Corporation, Armonk, NY, USA).

## Results

### Participants characteristics

A total of 1,600 GPs were enrolled, mostly female (61%; *n* = 976), aged between 30 to 39 years old (33%; *n* = 530) and practicing in European countries (68%; *n* = 1,081). Most of them had more than 20 years of experience as a GP (31%; *n* = 431), worked a median of 36 h per week (P25–P75: 28–40), in an urban setting (73%, *n* = 1,354) and were involved in teaching activities (64%; *n* = 1,017). Most of them had access to EHRs (95%, *n* = 1,523), were using it every day (91%, *n* = 1,379) and for more than 10 years (55%, *n* = 838). The characteristics of the participants are summarized in Table 1.

**Table 1:** Participants characteristics (*n* = 1,600).

**Characteristics**	**Total** **(*n* = 1,600)**
**Gender** ^a^	
Female	976 (61%)
Male	613 (39%)
**Age** ^b^	
<30 years	101 (6%)
30–39 years	530 (33%)
40–49 years	414 (26%)
50–59 years	325 (20%)
60–69 years	208 (12%)
70+ years	18 (1%)
**Country** ^c^	
European	1,081 (68%)
Non-European	517 (32%)
**Years of experience as GP**	
<5 years	335 (21%)
5–10 years	360 (23%)
10–15 years	241 (15%)
>15 years	173 (42%)
Hours of clinical work per week, median (P25–P75), hours^c^	36 (28–40)
**Setting of practice** ^c^	
Urban	1,354 (73%)
Rural	1,000 (63%)
Involvement in teaching^d^	1,017 (64%)
Access to EHRs^c^	1,523 (95%)
**Duration of use of EHRs** ^e^	
Only after COVID-19 outbreak	23 (2%)
Before COVID-19 outbreak, but <2 years	111 (7%)
(2–5) years	205 (14%)
(5–10) years	336 (22%)
>10 years	838 (55%)
**Frequency of access to EHRs** ^e^	
Less than 1^*^month	29 (2%)
At least 1^*^month	12 (1%)
At least 1^*^ week	27 (2%)
More than 1^*^ week	66 (4%)
Every day	1,379 (91%)

Unless otherwise indicated, values are displayed in *n* (%).

GP, General Practitioner; P25–P75, percentile 25 to percentile 75.

EHRs, electronic health records.

a Eleven GPs with missing information.

b Four GPs with missing information.

c Two GPs with missing information.

d Fifteen GPs with missing information.

e Eighty-seven GPs with missing information.

### Digital maturity and participants' characteristics

**Figure 2:**
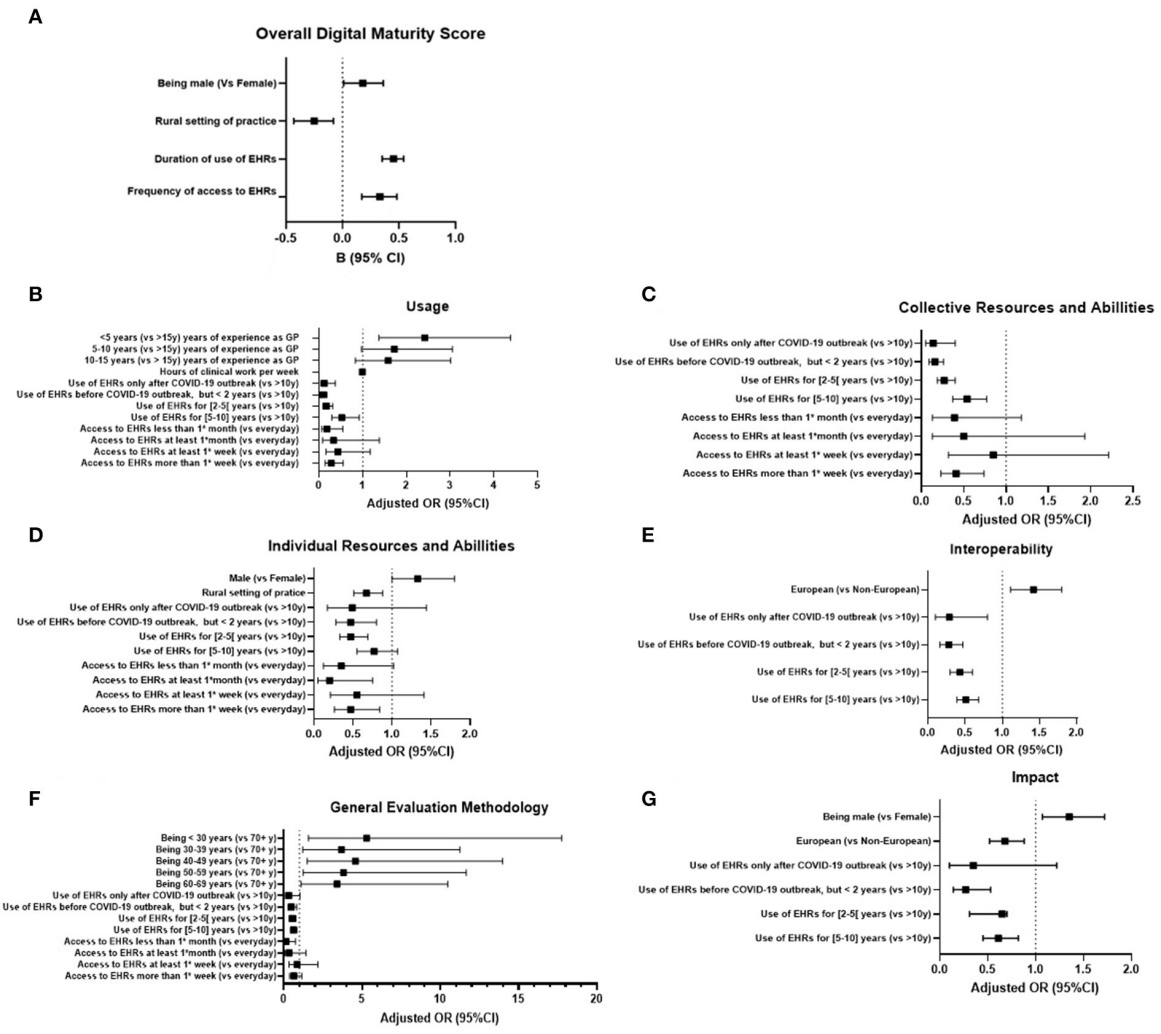
Forest plots of multivariable analysis for characteristics associated with overall digital maturity **(A)** and its dimensions-usage **(B)**, collective resources and abilities **(C)**, individual resources and abilities **(D)**, interoperability **(E)**, general evaluation methodology **(F)** and impact **(G)**. *B*, beta; OR, odds ratio; CI, confidence interval; vs, in comparison with.

Participants had a median digital maturity score of 4 (3–5). The highest three levels of the score accounted for almost 60% of the answers. Among the six dimensions, usage registered the highest percentage of agreement (90%, *n* = 1,209), followed by collective and individual resources and ability (80%, *n* = 1,073 and 77%, *n* = 1,035, respectively), impact (59%, *n* = 788) and interoperability (47%, *n* = 633). Best practice general evaluation methods registered the lowest scores of agreement (28%, *n* = 380). A significant multivariable linear regression model explained the digital maturity score (*R*^2^ = 11%, *P* < 0.001). Being male was associated with a higher digital maturity score [*B* = 0.18 (95% CI 0.01; 0.36)], while practicing in a rural setting was inversely associated with it [*B* = −0.25 (95% CI −0.43; −0.08)]. Additionally, longer duration and higher frequency of use of EHRs were also associated with a higher digital maturity score [*B* = 0.45 (95% CI 0.35; 0.54), *B* = 0.33 (95% CI 0.17; 0.48), respectively]. A detailed overview of the model is provided in Table 2 and a graphic representation in Figure 2A.

**Table 2:** Univariate and multivariable linear regression models to explain the digital maturity score.

**Characteristics**	**Univariate analysis**		**Multivariable analysis**	
	***B*** **(95% CI)**	***P*** **-value**	***B*** **(95% CI)**	***P*** **-value**
Gender (Ref = Female)	0.27 (0.08; 0.45)	0.005	0.18 (0.01; 0.36)	0.042
Age	0.18 (0.11; 0.26)	<0.001		
Country (Ref = Non-European)	0.26 (0.07;0.45)	0.008		
Years of experience as GP	0.21 (0.13;0.28)	<0.001		
Hours of clinical work per week	−0.01 (−0.01; 0.01)	0.864		
Rural setting of practice	−0.15 (−0.34; 0.40)	0.114	−0.25 (−0.43; −0.08)	0.005
Urban setting of practice	−0.03 (−0.28; 0.22)	0.797		
Involvement in teaching activities	0.19 (−0.01; 0.38)	0.056		
Access to EHRs	0.28 (−0.18; 0.74)	0.229		
Duration of use of EHRs	0.53 (0.44; 0.61)	< 0.001	0.45 (0.35; 0.54)	<0.001
Frequency of access to EHRs	0.57 (0.42; 0.72)	< 0.001	0.33 (0.17; 0.48)	<0.001

Ref, reference; B, unstandardized regression coefficient; 95% CI, 95% confidence interval; GP, General Practitioner; EHRs, electronic health record.

### Individual dimensions of digital maturity and participants' characteristics

Unadjusted ORs estimating the association between the characteristics of the participants and each of the six dimensions of the digital maturity are presented in Supplementary Table S2. Urban setting of practice was not associated with any dimension, while duration of use of EHRs was associated with all of them.

Adjusted ORs (aORs) represent the multivariable analysis of the predictors of each dimension and are summarized in Supplementary Table S3. The models explained 19% of the variance of usage, 13% of collective resources and ability, 6% of individual resources and ability, 7% of interoperability, 4% of general evaluation methods and 6% of impact. Hosmer Lemeshow tests showed that the models adequately fitted the data (*P* = 0.713, *P* = 0.983, *P* = 0.276, *P* = 0.554, *P* = 0.981, and *P* = 0.956, respectively).

#### Usage

GPs were less likely to use digital systems if they were using EHRs for a shorter period of time (aOR from 0.09 to 0.52) when compared to GPs accessing them for more than 10 years. Lower frequencies of access to EHRs were also associated with lower odds of use of the digital systems (aOR from 0.18 to 0.43) when compared to accessing them every day. On the other hand, in comparison with GPs practicing for more than 15 years, GPs who started practicing more recently had higher odds of using the digital systems (aOR from 1.58 to 2.42). The number of hours GPs worked in a week were negatively associated with usage of digital technologies [aOR = 0.99 (0.98; 1.00); Figure 2B].

#### Collective resources and ability

When compared to GPs accessing EHRs for more than 10 years, GPs who started accessing them later were less likely to express having collective resources and abilities (aOR from 0.14 to 0.54), as well as GPs who access EHRs less frequently (aOR from 0.39 to 0.85) when compared to GPs accessing them every day (Figure 2C).

#### Individual resources and ability

Being male was positively associated with reporting individual resources and ability [aOR 1.33 (95% CI 1.00; 1.80)], while practicing in a rural setting was negatively associated with it [aOR 0.67 (95% CI 0.51; 0.88)]. GPs who started accessing EHRs more recently were less likely to acknowledge individual resources and abilities (aOR from 0.47 to 0.77), when compared to GPs accessing them for more than 10 years. GPs who accessed EHRs less frequently were also less likely to acknowledge individual resources and ability (aOR from 0.20 to 0.55) when compared to GPs accessing them every day (Figure 2D).

#### Interoperability

In comparison with non-European GPs, Europeans were more likely to identify interoperability in the digital system they used [aOR = 1.42 (1.11; 1.80)]. In contrast, GPs who started accessing EHRs more recently were less likely to identify interoperability (aOR from 0.28 to 0.51) than those who have been accessing them for more than 10 years (Figure 2E).

#### General evaluation methods

Being European was associated with lower odds of practicing the best digital systems evaluation methods [aOR 0.68 (0.52; 0.88)]. Likewise, having started to access EHRs more recently was associated with lower odds of having best practice evaluation methods in place (aOR from 0.27 to 0.65; Figure 2F).

#### Impact

Males had higher odds of reporting digital system's impact (aOR1.35), as well as younger GPs (aOR 3.41–5.30) when compared to being 70 or more years old. On the other hand, in comparison with GPs who started to access EHRs over than 10 years ago, GPs who started accessing them more recently were associated with lower odds of recognizing impact of the digital systems they used (aOR from 0.33 to 0.62). Similarly, when compared to GPs with every day access to EHRs, GPs with less frequent accesses were less likely to identify impact as an asset of the digital systems (aOR from 0.16 to 0.86; Figure 2G).

## Discussion

### Principal findings

GPs had an overall good digital maturity score. While overall usage was the most acknowledged dimension of the digital maturity evaluation framework (90%), interoperability (47%) and use of best practice evaluation methods (28%) were dimensions which received a lower score, highlighting the potential for improvement in these areas.

Being male, having used EHRs for longer periods of time, and higher frequency of access to EHRs, were all positively associated with self-reported digital maturity. On the other hand, practicing in rural settings was negatively associated with digital maturity. No significant associations were found with age, country, years of experience as GP, hours of clinical work per week, urban setting of practice, involvement in teaching activities, and having or not access to EHRs.

All six dimensions of digital maturity may be explained by distinct characteristics, with shorter durations of use of EHRs being negatively associated with all of them.

### Comparison with previous literature

There has been an increase in the number of studies focused on developing digital maturity evaluation tools (28, 29). Although a considerable amount of research on this topic has been recently published, to our knowledge, there are no studies reporting the usage of such tools in primary care.

The World Health Organization has already recognized investment in resources, strategies for maximizing impact, standardized evaluation metrics and interoperability of systems, as key to the success of digital transformation (30). Interestingly, we found interoperability and general evaluation models to be the most prevalent shortcomings of digital systems maturity. Previous evidence regarding the determinants of digital health transformation in integrated care in Europe showed that although the importance of interoperability is well understood, the maturity of its implementation at present remains poor (31), a finding which is consistent with our findings. However, comparisons between studies should be interpreted with caution given the different tools used to assess digital maturity.

O'Donnel et al. conducted a systematic review on GPs attitudes toward EHRs which included 33 articles based on the American, European and Asian countries. It is concluded that the perception that EHRs can improve patient safety and quality of care common among GPs. Nevertheless, concerns regarding the impact of adynamic, rigid functionalities of EHRs in GPs' productivity were also raised (32). These findings are congruent with ours, since interoperability and best evaluation methodology—a necessary tool to enable positive changes in the systems to be made—are highlighted as the most prevalent digital maturity shortcomings despite the good overall digital maturity score.

Previous studies on the analysis of digital maturity determinants in secondary care focused on investigating whether availability of resources was related to digital maturity. In hospitals, investment in hardware and software was positively associated with higher levels of digital maturity (33). However, the effects of demographic factors, practice characteristics and adoption of EHRs features on digital maturity is less well documented in the literature.

Zaresani and Scott (33) have suggested that physicians who used digital health technology were more likely to be male. In the present study, being male was positively associated with digital maturity, but this information should be interpreted with caution due to the possibility of the existence of other confounding factors.

Gheorghiu and Hagens conducted a study in Canada to study the adoption of interoperable EHRs across different jurisdictions. They concluded that jurisdictions where physicians accessed interoperable EHRs more often were also the ones where they have already been doing so for longer periods of time. The authors used the frequency of end users' access to EHRs as a method of gauging the systems' maturity (34). Corresponding in our study, GPs accessing EHRs more frequently were associated not only with higher overall digital maturity, but also with better scores on usage, collective resources and abilities, individual resources and abilities and impact of the digital systems they used. The duration of use of EHRs was also associated with better overall digital maturity and with each of its six dimensions.

Regarding clinical practice in rural areas, this was negatively associated with the maturity of digital systems. Although there was sparse evidence specifically exploring the impact of the practice setting on the digital maturity of health systems, existing studies noted that rural areas often remain left behind in terms of broadband coverage and other forms of digital connectivity, as well as lower rates of digital adoption and skills (35).

### Strengths and limitations

This study has several strengths. To the best of our knowledge, it is the first study focusing on the evaluation of digital maturity indicators across patient pathway in primary care and the exploitation of its determinants across distinct countries in the perspective of GPs. Participants were GPs working from 20 different countries worldwide, with diversified resource management policies in primary care. A comprehensive set of participants' demographic characteristics, practice characteristics and features of EHRs adoption was collected and analyzed, which allowed us to explore their role in digital maturity.

However, this study also has some limitations that should be acknowledged. It is based on a non validated questionnaire, which gives no guarantees that the collected variables are truly measuring digital maturity. The questionnaire was disseminated online *via* email and social media channels and therefore a potential selection bias cannot be excluded. For example, we can hypothesize that GPs that were more prone to answer the online questionnaire were those working with higher digital maturity. This can possibly explain that 55% of the participants were using EHRs for more than 10 years and 91% were accessing them every day. Additionally, the lack of translation of this questionnaire to the official languages of all 20 inSIGHT Research Group member countries might have presented an obstacle to its enrollment in certain countries. Nevertheless, this data collection methodology enabled us to gather data from 20 countries in a short period of time, proving it to be prompt, economical, and safe to use. Due to its cross-sectional design, this study only enabled us to assess digital maturity during a specific period. It would be important to reproduce this online questionnaire in the future, to allow deductions on the digital maturity temporal evolution to be made.

Additionally, it is important to stress that the framework used to assess digital maturity was developed in 2016 and the employment of digital technologies in health has been rapidly changing since then. However, this tool was a result of a systematic search about the best methods and metrics for evaluating digital maturity and allowed us to perform a patient-centric evaluation focused on identifying how digital maturity can be most significantly refined in the health sector. The choice of evaluating digital maturity at the primary care level only was made since the focus of our work was in fact general practice. Future studies should consider the utilization of the entire framework across four levels (home, community, primary and secondary care) since the evaluation of the digital maturity of health services is dependent on a sector-wide patient understanding (11).

Finally, most GPs included in this study were female (61%), European (68%), involved in teaching activities (64%). Therefore, any attempts to generalize these findings to populations with different characteristics need to be approached with caution.

### Implications for research and policy

Our study provides an initial overview of the factors that impact digital maturity and highlights discrepancies in digital transformation across healthcare settings. Future research should evaluate how specific characteristics and features of different healthcare systems, and countries, impact the various aspects of digital maturity and its overall score. Robust comparisons across countries will need to adequately adjust for these factors, and their potential impact as mediators or confounders to robustly support learning from best practices. Additionally, future research should address and measure, other aspects of digital maturity in primary care, beyond the scope of EHRs interoperability.

## Conclusion

This is the first international study performed in general practice providing important results for putting into practice in different levels. This work generates evidence on the level of digital maturity in primary care. It demonstrates interoperability and best practice evaluation methods of the digital systems as common digital maturity shortcomings in primary care, which prioritizes the need for these two dimensions to be addressed by stakeholders in order to improve digital maturity across health systems. Our results identified a negative association between practicing general medicine in a rural setting and the level of digital maturity, highlighting discrepancies across various healthcare settings which can slow overall digital transformation.

Therefore, our findings can help to inform key stakeholders in digital health, mainly to policymakers, in developing more bespoke and effective strategies to hasten and take the best advantage of the ongoing digital transformation in General Practice.

## Data availability statement

The original contributions presented in the study are included in the article/Supplementary material, further inquiries can be directed to the corresponding author.

## Ethics statement

The studies involving human participants were reviewed and approved by Imperial College Research Ethics Committee (Reference 20IC5956). The patients/participants provided their written informed consent to participate in this study.

## Author contributions

FT, CJ, and AN wrote the first manuscript. All authors reviewed the manuscript and approved the version submitted for publication.
