# Supplementary material for: Digital maturity and its determinants in General Practice: A cross-sectional study in 20 countries
Source: Front Public Health. 2023 Jan 13;10:962924. doi: 10.3389/fpubh.2022.962924 (PMC9880412; doi:10.3389/fpubh.2022.962924)
Supplement: Supplementary file 1 [file Table_1.docx]

Supplementary Material

**SUPPLEMENTARY FILES:**

Supplementary Table 1 – Full list of question asked to the study participants and respective options of answer.

| Variable | Survey question | Categories |
| --- | --- | --- |
| Gender | Please select your gender | Female  Male  Other  Prefer not to answer |
| Age | Please select your age from the categories below: | Under 30  30-39  40-49  50-59  60-69  70+  Prefer not to answer |
| Country | In which country do you currently practice? | Australia  Brazil  Canada  Chile  Colombia  Croatia  Finland  France  Germany  Ireland  Israel  Italy  Poland  Portugal  Slovenia  Spain  Sweden  Turkey  United Kingdom  United States |
| Practice setting | In which type of setting is your practice located? | Urban  Rural  Mixed |
| Number of hours of clinical work per week, | On average, how many hours of clinical work do you have per week? | [Free text] |
| Number of years of experience as GP | For how long have you been working as a General Practitioner/Family Doctor? | < 5 years  5 - 10 years  10 - 15 years  15 - 20 years  > 20 years |
| Involvement in teaching activities | Are you involved in teaching activities? (i.e. teaching trainees or medical students, or affiliated to a university) | Yes  No  Prefer not to answer |
| Availability of EHRs, | In your practice, do you have access to electronic health records? | Yes  No |
| Duration of use | For how long have you been using an electronic health record in your practice? | Only after the COVID-19 outbreak Before COVID-19 outbreak, but for less than 2 years  2-5 years  5-10 years  More than 10 years |
| Frequency of use | How often do you personally access electronic health records in your practice? | Less than once a month  At least once a month  At least once a week  More than once a week  Everyday |
| Digital maturity (Usage) | In order to help us understand the digital maturity of your electronic health record system, please tell us how much you agree with the following statement:  *Most healthcare providers in our practice use the digital system* | Agree  Neutral  Disagree |
| Digital maturity (Resources & ability - organisational) | In order to help us understand the digital maturity of your electronic health record system, please tell us how much you agree with the following statement:  *Our organisation is ready to use the digital system correctly* | Agree  Neutral  Disagree |
| Digital maturity (Resources & ability - individual) | In order to help us understand the digital maturity of your electronic health record system, please tell us how much you agree with the following statement:  *We have the individual abilities needed to use the digital system correctly* | Agree  Neutral  Disagree |
| Digital maturity (Interoperability) | In order to help us understand the digital maturity of your electronic health record system, please tell us how much you agree with the following statement:  *Our digital system has the capability to communicate across services or with other systems* | Agree  Neutral  Disagree |
| Digital maturity (General evaluation methodology) | In order to help us understand the digital maturity of your electronic health record system, please tell us how much you agree with the following statement:  *We have best practice digital maturity evaluation methods in place* | Agree  Neutral  Disagree |
| Digital maturity (Impact) | In order to help us understand the digital maturity of your electronic health record system, please tell us how much you agree with the following statement:  *Our system has a positive impact in terms of outcomes for patients, structure, process or finance* | Agree  Neutral  Disagree |
