# Supplementary material for: Digital maturity and its determinants in General Practice: A cross-sectional study in 20 countries
Source: Front Public Health. 2023 Jan 13;10:962924. doi: 10.3389/fpubh.2022.962924 (PMC9880412; doi:10.3389/fpubh.2022.962924)
Supplement: Supplementary file 3 [file Table_3.docx]

Supplementary Material

**SUPPLEMENTARY FILES:**

Supplementary Table 3 - Multivariable logistic regression models to explain each dimension of the framework: usage, collective resources and ability, individual resources and ability, interoperability, general evaluation and impact. Reference – the category used as reference. OR – Odds Ratio; 95%CI - 95% Confidence Interval; GP- General Practitioner, EHRs - Electronic Health Records

|  |  | Usage | | Collective Resources and ability | | Individual Resources and ability | | Interoperability | | General Evaluation Methods | | Impact | |
| --- | --- | --- | --- | --- | --- | --- | --- | --- | --- | --- | --- | --- | --- |
| Characteristics | | OR [95% CI] | P  value | OR [95%CI] | P value | OR [95% CI] | P value | OR [95% CI] | P  value | OR [95% CI] | P  value | OR [95% CI] | P  value |
|  | |  |  |  |  |  |  |  |  |  |  |  |  |
| Gender | |  |  |  |  |  |  |  |  |  |  |  |  |
|  | Male |  |  |  |  | 1.33 [1.00;1.80] | 0.047 |  |  |  |  | 1.35 [1.07;1.72] | 0.013 |
|  | Female | Reference |  |  |  |  |  |  |  |  |  |  |  |
| Age | |  |  |  |  |  |  |  |  |  |  |  |  |
|  | < 30 years |  |  |  |  |  |  |  |  |  |  | 5.30 [1.58;17.76] | 0.007 |
|  | 30-39 years |  |  |  |  |  |  |  |  |  |  | 3.69 [1.21;11.26] | 0.022 |
|  | 40-49 years |  |  |  |  |  |  |  |  |  |  | 4.58 [1.50;13.97] | 0.007 |
|  | 50-59 years |  |  |  |  |  |  |  |  |  |  | 3.82 [1.25;11.66] | 0.019 |
|  | 60-69 years |  |  |  |  |  |  |  |  |  |  | 3.41 [1.11;10.48] | 0.032 |
|  | 70+ years | Reference |  |  |  |  |  |  |  |  |  |  |  |
| Country | |  |  |  |  |  |  |  |  |  |  |  |  |
|  | European |  |  |  |  |  |  | 1.42 [1.11;1.80] | 0.003 | 0.68 [0.52;0.88] | 0.005 |  |  |
|  | Non-European | Reference |  |  |  |  |  |  |  |  |  |  |  |
| Years of experience as GP | |  |  |  |  |  |  |  |  |  |  |  |  |
|  | <5 years | 2,42 [1.37;4.38] | 0.003 |  |  |  |  |  |  |  |  |  |  |
|  | 5-10 years | 1.72 [0.97;3.05] | 0.063 |  |  |  |  |  |  |  |  |  |  |
|  | 10-15 years | 1.58 [0.83;3.01] | 0.161 |  |  |  |  |  |  |  |  |  |  |
|  | >15 years | Reference |  |  |  |  |  |  |  |  |  |  |  |
| Hours of clinical work per week | | 0.99 [0.98;1.00] | 0.022 |  |  |  |  |  |  |  |  |  |  |
| Rural setting of practice | |  |  |  |  |  |  |  |  |  |  |  |  |
|  | Yes |  |  |  |  | 0.67 [0.51;0.88] | 0.004 |  |  |  |  |  |  |
|  | No | Reference |  |  |  |  |  |  |  |  |  |  |  |
| Duration of use of EHRs | |  |  |  |  |  |  |  |  |  |  |  |  |
|  | Only after COVID-19 outbreak | 0.12 [0.04;0.37] | <0.001 | 0.14 [0.05;0.40] | <0.001 | 0.49 [0.17;1.44] | 0.195 | 0.29 [0.10;0.80] | 0.018 | 0.35 [0.10;1.22] | 0.099 | 0.33 [0.11;1.04] | 0.059 |
|  | Before COVID-19 outbreak.  but < 2 years | 0.09 [0.04;0.18] | <0.001 | 0.16 [0.09;0.26] | <0.001 | 0.47 [0.28;0.80] | 0.005 | 0.28 [0.16;0.47] | <0.001 | 0.27 [0.14;0.53] | <0.001 | 0.49 [0.28;0.83] | 0.008 |
|  | [2-5[ years | 0.17 [0.09; 0.31] | <0.001 | 0.27 [0.19;0.40] | <0.001 | 0.47 [0.33;0.69] | <0.001 | 0.43 [0.30;0.60] | <0.001 | 0.65 [0.31;0.70] | <0.001 | 0.54 [0.37;0.79] | 0.001 |
|  | [5-10] years | 0.52 [0.29;0.92] | 0.028 | 0.54 [0.37:0.77] | 0.001 | 0.77 [0.55;1.07] | 0.119 | 0.51 [0.39;0.68] | <0.001 | 0.61 [0.45;0.82] | 0.001 | 0.62 [0.46;0.84] | 0.002 |
|  | > 10 years | Reference |  |  |  |  |  |  |  |  |  |  |  |
| Frequency of access to EHRs | |  |  |  |  |  |  |  |  |  |  |  |  |
|  | Less than 1* month | 0.18 [0.06;0.54] | 0.002 | 0.39 [0.13;1.18] | 0.095 | 0.35 [0.12;1.02] | 0.054 |  |  |  |  | 0.16 [0.03;0.75] | 0.020 |
|  | At least 1*month | 0.33 [0.08;1.38] | 0.130 | 0.50 [0.13;1.93] | 0.315 | 0.20 [0.05;0.75] | 0.017 |  |  |  |  | 0.35 [0.0.9;1.42] | 0.141 |
|  | At least 1* week | 0.43 [0.16;1.17] | 0.098 | 0.85 [0.32;2.21] | 0.732 | 0.55 [0.21;1.41] | 0.211 |  |  |  |  | 0.86 [0.34;2.19] | 0.755 |
|  | More than 1* week | 0.28 [0.14;0.55] | <0.001 | 0.41 [0.23;0.74] | 0.003 | 0.47 [0.26;0.84] | 0.011 |  |  |  |  | 0.66 [0.38;1.16] | 0.153 |
|  | Everyday | Reference |  |  |  |  |  |  |  |  |  |  |  |
